# The relative importance of direct and indirect effects of hunting mortality on the population dynamics of brown bears

**DOI:** 10.1098/rspb.2014.1840

**Published:** 2015-01-07

**Authors:** Jacinthe Gosselin, Andreas Zedrosser, Jon E. Swenson, Fanie Pelletier

**Affiliations:** 1 Département de biologie, Université de Sherbrooke, 2500 boulevard de l'Université, Sherbrooke, Quebec, Canada J1K 2R1; 2 Department of Environmental and Health Studies, Telemark University College, Bø 3800, Norway; 3 Institute of Wildlife Biology and Game Management, University of Natural Resources and Life Sciences, Vienna 1180, Austria; 4 Department of Ecology and Natural Resource Management, Norwegian University of Life Sciences, Ås 1432, Norway; 5 Norwegian Institute for Nature Research, Trondheim 7485, Norway

**Keywords:** population dynamics, harvesting, brown bear, sexually selected infanticide, behaviour, carnivore

## Abstract

There is increasing evidence of indirect effects of hunting on populations. In species with sexually selected infanticide (SSI), hunting may decrease juvenile survival by increasing male turnover. We aimed to evaluate the relative importance of direct and indirect effects of hunting via SSI on the population dynamics of the Scandinavian brown bear (*Ursus arctos*). We performed prospective and retrospective demographic perturbation analyses for periods with low and high hunting pressures. All demographic rates, except yearling survival, were lower under high hunting pressure, which led to a decline in population growth under high hunting pressure (*λ* = 0.975; 95% CI = 0.914–1.011). Hunting had negative indirect effects on the population through an increase in SSI, which lowered cub survival and possibly also fecundity rates. Our study suggests that SSI could explain 13.6% of the variation in population growth. Hunting also affected the relative importance of survival and fecundity of adult females for population growth, with fecundity being more important under low hunting pressure and survival more important under high hunting pressure. Our study sheds light on the importance of direct and indirect effects of hunting on population dynamics, and supports the contention that hunting can have indirect negative effects on populations through SSI.

## 1. Introduction

Understanding the population dynamics of exploited species is essential to determine sustainable harvest rates for wildlife populations. Harvesting individuals obviously can have important direct effects on the growth rate of a population by increasing mortality rates. However, there is increasing evidence that harvesting also can have indirect effects on population growth [1]. For instance, harvest can disrupt the sex and age structure of a population, which can in turn affect fecundity rates [1–3].

Harvesting may also have an indirect effect on populations by affecting behaviour [4]. Individual behaviour is now considered to be an important factor influencing population dynamics [5,6]. Any individual behaviour that influences reproductive success and survival should also influence population growth. For example, hunting has been shown to affect individual movement rates in elk (*Cervus elaphus*) [7,8], activity patterns in brown bears (*Ursus arctos*) [9], and habitat selection in wild boar (*Sus scrofa*) [10] and mule deer (*Odocoileus hemionus*) [11]. As changes in behavioural patterns caused by hunting may affect food intake, it has the potential to affect the survival and fecundity of individuals.

Harvesting can also affect the expression of certain behaviours in surviving individuals. For example, harvesting is thought to increase the rate of social reorganization in some species, which promotes male turnover and new encounters between individuals, thus leading to an increase of sexually selected infanticide (SSI) [12,13]. SSI occurs when competition between members of one sex for the other sex may make it advantageous for an individual (usually a male) to eliminate offspring of another individual [14]. SSI occurs in a wide array of species, including Rodentia (see [15] for review), non-human primates (e.g. Hanuman langur *Presbytis entellus* [16,17]; but see also [18]) and carnivores [19]. Carnivores are often hunted, with harvest generally focused on males, particularly when they are hunted for trophies [20,21]. In species with SSI, harvesting males can have an indirect negative effect on the population by reducing juvenile survival [4,21].

Although several studies have quantified how behaviour can affect reproductive success and survival [22–24], only a few have linked behaviour to population dynamics [4,25]. The influence of behaviour on population dynamics and its interaction with harvest is difficult to quantify in long-lived wild species, as it requires long-term data on the survival, reproduction and behaviour of individuals as well as on population dynamics [25]. The goal of this study was to assess the direct and indirect effects of hunting through SSI on the dynamics of a brown bear (*U. arctos*) population.

To evaluate the influence of hunting and SSI on population dynamics, we performed prospective and retrospective perturbation analyses for periods with different hunting pressures. Our goals were to determine how demographic rates and population growth vary under low and high hunting pressure and to determine the relative importance of demographic rates, including cub survival, on population growth. We predicted (P1) that hunting would have a direct negative effect on population growth by reducing the survival rates of age classes available for hunting. We also expected (P2) that hunting would have an indirect negative effect on population growth through SSI, owing to lower cub survival. Further, we predicted (P3) that cub survival would have a lower elasticity (i.e. relative influence on population growth) than most other demographic rates, using the prospective analyses [26]. As demographic rates with low elasticity, such as juvenile survival, usually have high variability [26], we predicted (P4) that cub survival would explain a substantial proportion of the variation in population growth using the retrospective analyses. Thus, by evaluating the importance of cub survival, a proxy of SSI, for population growth, we aimed to better understand the effects of behaviour on the population dynamics of a long-lived wild mammal species. We hoped that the results would increase our understanding of both the direct and indirect effects of hunting on population dynamics.

The Scandinavian brown bear offers a unique opportunity to evaluate not only the direct effects of hunting, but also the potential indirect effects that hunting may have on population dynamics through behaviour. SSI is common in Scandinavian brown bears [12,27,28], although its occurrence in North American brown bear populations is controversial [29,30] (but see also [31]). Nevertheless, the species has characteristics that should promote SSI [19]. The long period of maternal care (between 1.5 and 4.5 years) reduces the availability of reproductive females and a female may become receptive only 2–4 days after losing her young during the mating season [32–34]. Therefore, males would benefit from killing cubs of the year (hereafter referred to as cubs) during the mating season [28,34]. Swenson *et al.* [35] found that 85% of the mortality of cubs occurs during the mating season in Scandinavia, and all confirmed cub mortalities during the mating season were cases of infanticide (14 cubs in 2009–2011) [27]. There is therefore strong evidence of SSI in the Scandinavian brown bear population [28] and it seems to greatly affect cub survival. Moreover, brown bears are hunted in Scandinavia and there is evidence that SSI might increase with hunting pressure [12,35,36]. Indeed, cub survival is lower (from 28% to 42%) when at least one male had been killed in the same area 0.5, and especially 1.5, years earlier [12]. This cub mortality is thought to be caused by SSI, which is promoted by the male turnover created when males die during the hunting season [12,35–37]: when a resident male is killed, he will be replaced by a male who is probably unrelated to cubs present in the area, thus leading to an increase in SSI [12,13].

## 2. Methods

### (a) Study area and population

The study area was located in southcentral Sweden (61° N, 15° E), mostly in the counties of Dalarna and Gävleborg. It is composed of 13 000 km² of rolling landscape (from 200 to 1000 m) with intensively managed boreal forest dominated by Scots pine (*Pinus sylvestris*) and Norway spruce (*Picea abies*) [38]. The Scandinavian population is one of the most productive brown bear populations in the world [39], with an early mean age at first reproduction (4.71 years [36]) and short interlitter intervals (1.6 years [35]). Density of bears in the study area increased over our study period (1990–2011), although not evenly nor constantly [40,41]. Demographic consequences of this increase are unknown, but they are unlikely to affect subadult and adult survival [42]. Indeed, hunting is the main cause of mortality for bears aged 1 year and older, and 84.4% of deaths of marked bears in our study area were caused by humans from 1990 to 2011. Most natural mortalities are intraspecific predation and affect mostly yearlings and subadults [43]. Another study has also suggested that the population did not seem to be food limited [40]. Therefore, hunting is the main driver of the population and fluctuations in harvest rates explain 83% of the population trend [44].

### (b) Data collection

#### (i) Captures and monitoring

Females without young and females accompanied by yearlings were immobilized with a dart gun from a helicopter. Captures were carried out after den emergence from mid-April to early May. Females with cubs were not captured for animal welfare reasons. All females were marked individually with tattoos (inside the upper lip), and passive integrated transponder (PIT) tags under anaesthesia. Females were fitted with radiotransmitters, radio-implants (Telonics, model IMP/40/L HC), or both. Females were originally fitted with VHF radiotransmitters (Telonics, model 500). However, since 2003, most (gradually from 6% to 90%) females captured or recaptured were fitted with GPS–GMS transmitters (GPS Plus, Vectronic Aerospace GmbH). A vestigial premolar tooth was collected from all females not captured as a yearling to estimate age based on the cementum annuli in the root (Mattson's Inc., Milltown, MT). For further information about capture and handling of bears, see Arnemo *et al.* [45] and Zedrosser *et al.* [46].

Females fitted with VHF radiotransmitters were located once a week during the non-denning period using standard triangulation methods [47]. Females fitted with GPS radiotransmitters were located at least once every 30 min during the active period. To ascertain timing of cub loss, females with cubs were observed from a helicopter three times per year: at den emergence (early May), after the breeding season (mid-July) and in autumn before den entrance (late September to early October). Most cub loss (80.9%) occurred during the breeding season (mid-May to mid-July). Litter size was defined as the number of cubs observed with the mother at the first sighting following den emergence.

#### (ii) Hunting

Bears were hunted across the entire study area. Hunting started in late August or early September and lasted until either 15 October or when the quota within the designated area had been reached, whichever came first. Hunters could kill any solitary bear, regardless of sex and age. The only protected segment of the population was family groups (i.e. females and their dependent offspring of any age) [48].

After harvesting a bear, hunters were required to report the kill and present the carcass to an official inspector on the same day. Hunters were required to give information about hunting method, sex of the bear, body weight and the location of the harvest. In addition, hunters provided a premolar tooth for age determination. The sex ratio of individuals harvested was 45% female and 55% male. The Swedish bear hunt and reporting of hunter-killed bears are further described by Bischof *et al.* [48].

### (c) Statistical analyses

#### (i) Subperiods of consistent hunting pressure

As demographic models are better performed on relatively long periods of time, we tested the effect of hunting on the population by comparing the population dynamics in periods of different hunting pressure. We calculated the yearly hunting pressure in our study area as the number of marked bears that had been killed legally divided by the number of marked bears available for hunting (i.e. the number of marked bears known to be alive at the start of the hunting season, excluding family groups). We tested whether there were periods with statistically different hunting pressure over the study period by dividing the study period into 2–5 subperiods and calculating the Calinski–Harabasz (CH) index for all possible chronological combinations of subperiods. The CH index is computed as [trace *B*/(*k −* 1)]/[trace *W*/(*n − k*)], where *n* and *k* are the total number of items and the number of clusters in the solution, respectively. The *B* and *W* terms are the between- and within-cluster sum of squares and cross product matrices, and the trace is the sum of the main diagonal of the matrices [49,50]. Higher values of the CH index represent higher between-cluster variance relative to within-cluster variance. We compared the CH index for the most probable chronological groups and determined the most likely number of subperiods. The maximum hierarchy level was used to indicate the correct number of partitions in the data, which maximized between-cluster variance and minimized within-cluster variance.

#### (ii) Demographic parameters

We modelled only the female component of the population, because in brown bears, as in most large mammals, it is the number of reproductive females that limits reproduction [51,52]. To ascertain which age classes best represented the life stages in the population, we tested different age-class models and selected the one that best described the survival pattern. Model selection was based on Akaike's information criterion corrected for small sample sizes (AICc) [53].

The recapture probability of females alive in the study area was estimated to be 100% [43]. Therefore, survival and reproductive output of the females were assessed from repeated observations of the individuals. Based on these data, we calculated the mean survival and fecundity for each age class over the study period and for each subperiod. Fecundity rates represent the probability that a female produces a cub the following year (fecundity*_t_*_→_ *_t_*_+1_ = survival*_t_*_→_ *_t_*_+1_ × reproduction*_t_*_→_ *_t_*_+1_). The demographic rates were calculated from all of the survival and reproduction information available from the females followed during 1990–2011. We lost contact with some females (about 14%) without known mortality. A sensitivity analysis revealed that whether or not we included individuals with truncated life histories did not affect demographic rates (see the electronic supplementary material, table S1). Therefore, we included individuals with unknown mortality for the period they were followed. Demographic rates were used to construct pre-breeding quasi-Leslie matrices describing the transition probabilities between or within age classes from one year to the next [54]. One matrix was built for the entire study period, and other matrices were built on subsets of the data corresponding to each hunting pressure subperiod (1990–2005 and 2006–2011; see Results). Because cubs were not captured, their sex was therefore unknown. All cubs were used for cub survival and fecundity estimations. We assumed that there was no difference in survival between male and female cubs, which has been suggested in our population [55]. Fecundity rates were adjusted using a secondary sex ratio of 50 : 50 [56].

#### (iii) Prospective analysis

Prospective analyses predict the change in the asymptotic growth rate that would result from a change in a demographic rate and are independent of past variation in demographic rates [57]. We calculated the asymptotic growth rate of the population (*λ*, the exponential growth rate at the stable age distribution) for the entire study period and for each hunting pressure subperiod. We calculated elasticities of the population growth rate independently from each matrix for each demographic rate. Elasticities of the population growth are the proportional change in *λ* resulting from a proportional change in a demographic rate (*r_i_*), Δlog*λ*/Δlog *r_i_* [54]. Prospective analyses were performed with the ‘popbio’ package in R [58]; the confidence intervals of *λ* were calculated with the ‘boot.transitions’ function.

#### (iv) Retrospective analysis

Retrospective analyses compare the contributions of past changes in demographic rates with the variation in *λ* and are not indicative of future changes [57]. We estimated the association between variation in a demographic rate *r_i_* and variation in *λ* by: 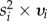, where *s_i_* is the sensitivity of the population growth rate to a demographic rate *r_i_*, and *υ_i_* is the variance of *r_i_* [59]. These associations are presented as contributions to variation in *λ*, when rescaled as percentages. We did not include covariations of demographic rates in the analysis, owing to low annual sample size. Calculations and statistics were performed using R v. 3.0.0 [60].

## 3. Results

### (a) Subperiods of consistent hunting pressure

**Figure 1.**
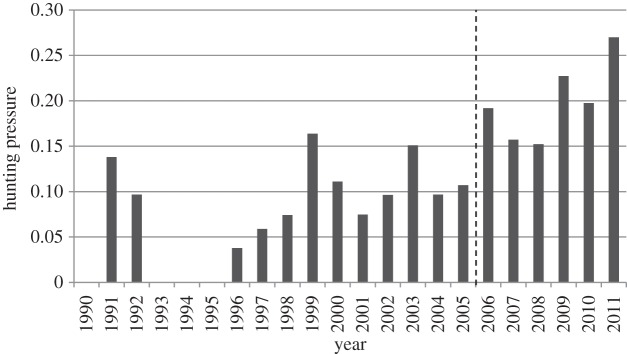
Hunting pressure (the number of marked bears that were legally killed divided by the number of marked bears available for hunting; see Methods) on brown bears in southcentral Sweden from 1990 to 2011. There were two subperiods with different hunting pressures: 1990–2005 (low) and 2006–2011 (high) (see the electronic supplementary material, table S2). The dashed line separates the two hunting pressure subperiods.

Based on the highest CH index, the most likely number of subperiods with different levels of hunting pressure was two (see the electronic supplementary material, table S2). The two subperiods that minimized intragroup variation and maximized intergroup variation were 1990–2005, with low hunting pressure (0.073 ± 0.014, mean ± s.e.; figure 1), and 2006–2011, with high hunting pressure (0.199 ± 0.018; figure 1). Consequently, we retained these two periods in our subsequent analyses. The sex ratio of bears harvested changed slightly between the two hunting pressure subperiods (48% females, 52% males in 1990–2005 versus 43% females, 57% males in 2006–2011; Yates *χ*² = 3.97, *p*-value = 0.046).

### (b) Demographic rates

The model that best represented the life stages in the population identified six distinct age groups: 0-, 1-, 2- and 3-year-olds, young adults (4–8 years old) and older adults (9 years and older; electronic supplementary material, figure S1). Matrix dimensions were therefore 6 × 6. Details on model selection can be found in the electronic supplementary material, tables S3–S5.

**Figure 2.**
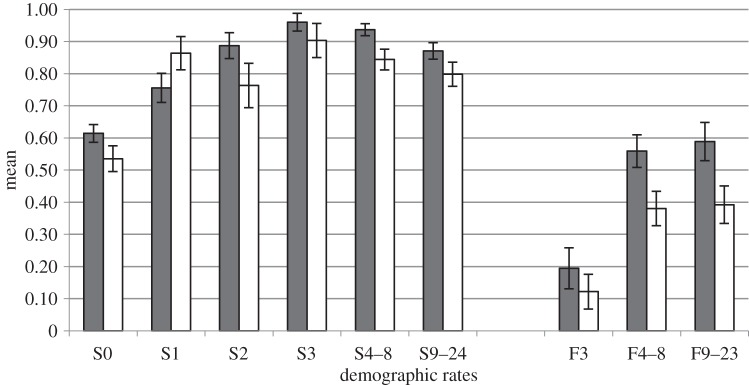
Means and standard errors of the survival (S) and fecundity (F) rates for different age classes (see text) of female brown bears in southcentral Sweden from 1990 to 2005 (grey bars) and 2006 to 2011 (white bars).

We estimated cub survival from 466 cubs born in 203 litters to 69 marked females between 1990 and 2011. Survival of females aged 1 year and older was estimated from 180 marked females of known age (*n* = 901 individual-years; for further information on sample size, see the electronic supplementary material, table S6). During the entire study period (1990–2011), mean cub survival was estimated at 58.8% and survival of females was highest at 3 years of age (table 1). In general, survival rates in the high harvest subperiod were lower than in the low harvest subperiod, with the exception of yearling survival, which was higher in the high harvest subperiod (figure 2).

**Table 1.** Means, standard errors, elasticities, variances and the retrospective analysis results of the demographic rates for different age classes of female brown bears in southcentral Sweden from 1990 to 2011. The results of the retrospective analysis give the proportion of the variation in *λ* that is explained by the variation in each demographic rate (y.o., years old).

demographic rate	mean	standard error	elasticity	variance	retrospective analysis (%)
cub survival	0.588	0.023	0.104	0.243	16.838
yearling survival	0.791	0.035	0.104	0.167	6.381
2 y.o. survival	0.840	0.037	0.104	0.136	4.613
3 y.o. survival	0.938	0.027	0.098	0.059	1.426
4–8 y.o survival	0.904	0.017	0.306	0.087	22.383
9–24 y.o survival	0.842	0.022	0.178	0.134	13.281
3 y.o. fecundity	0.166	0.044	0.006	0.281	0.745
4–8 y.o. fecundity	0.488	0.038	0.056	0.710	20.773
9–23 y.o. fecundity	0.502	0.042	0.042	0.868	13.559

We calculated fecundity from the reproduction of marked females 4–24 years old (*n* = 178 individuals; *n* = 493 individual-years, data from 1990 to 2011; for further information on sample size, see the electronic supplementary material, table S7). In the entire study period, fecundity was highest for females aged 9 years and older (table 1). Fecundity rates were lower in the high hunting pressure subperiod than in the low hunting pressure subperiod (figure 2).

### (c) Prospective analysis

For the entire period, the asymptotic growth rate (*λ*) of the population was 1.041 (95% CI = 1.012–1.069; see the electronic supplementary material, figure S2). The asymptotic population growth rate was higher in the low hunting pressure subperiod (*λ* = 1.082; 95% CI = 1.052–1.119; see the electronic supplementary material, figure S2) and was lower during the high hunting pressure subperiod (*λ* = 0.975; 95% CI = 0.914–1.011; see the electronic supplementary material, figure S2). Survival of adult females had the greatest elasticities (0.306 for young and 0.178 for old adults for the entire period; table 1), followed by the survival of juveniles, including cub survival (approx. 0.1; table 1). Elasticities of survival rates were greater than for the corresponding fecundity rates (table 1). Summed elasticities for female survival (0.894 for the entire study period) far exceeded elasticities for reproduction (0.104 for the entire study period). Elasticities of the demographic rates were qualitatively equivalent in the two different hunting pressure subperiods and were similar to those obtained in the global period (see the electronic supplementary material, table S8).

### (d) Retrospective analysis

**Figure 3.**
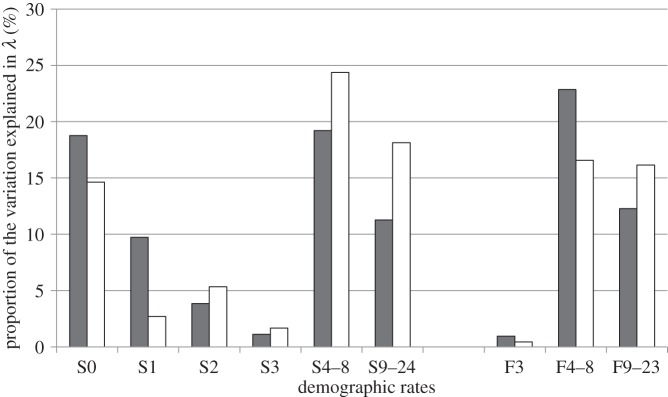
Proportion of the variation in *λ* (%) that is explained by the variation in survival (S) and fecundity (F) rates for different age classes (see text) of female brown bears in southcentral Sweden from 1990 to 2005 (grey bars) and 2006 to 2011 (white bars).

In all periods, the survival and fecundity of adult females explained the most variation in *λ* (table 1 and figure 3). In the global model and in the high hunting pressure subperiod, the survival of adult females explained the most variation in the growth rate (35.7% and 42.5%, respectively; table 1 and figure 3), followed by the fecundity of adult females (35.1% and 33.1%, respectively; table 1 and figure 3). In the low hunting pressure subperiod, however, the fecundity of adult females explained the most variation (36.1%), followed by their survival (30.5%; figure 3). Cub survival explained between 14.6% and 18.8% of the variation in population growth in the different models (table 1 and figure 3).

## 4. Discussion

The goal of this study was to quantify the direct and indirect effects of hunting on the population dynamics of a large long-lived mammal, the brown bear. Our analyses produced three main results. First, we found that adult females were the most important groups affecting population dynamics, having the highest elasticities and explaining the most variation in *λ*. Second, we found pronounced differences between the two subperiods with different hunting pressures: the demographic rates, including survival rates of age classes available for hunting and cub survival, were lower under high hunting pressure, leading to a decrease in *λ*, in accordance with P1 and P2. In addition, the relative contribution of survival and fecundity to the variance of *λ* changed with hunting pressure, with fecundity being more important under low hunting pressure and survival being more important under high hunting pressure. Third, we found that cub survival showed a relatively high importance for population growth (third highest elasticity, contrary to P3) and explained a substantial proportion of the variation in *λ* in the retrospective analyses (ranging from 14.6 to 18.8%) in accordance with P4.

Previous studies have revealed that survival of prime-aged females is the vital rate, with the highest elasticity in most large mammal populations (e.g. [26,52,61–64]). This pattern is expected in long-lived species, because higher adult survival leads to more reproductive opportunities. Our study also was consistent with this pattern, with survival rates of adult females having the highest elasticities and the variation in survival and fecundity rates of adult females explaining the largest proportion of the variation in *λ*. It has been suggested that there may be a trade-off between the intrinsic dependence of *λ* on a demographic rate and the degree of observed temporal variation in that demographic rate [26,65]. In fact, traits with the greatest potential impact on population growth tend to be under high selection and to have lower temporal variability [26,65]. Our results, however, suggest that when human-induced mortality is high (in Sweden, nearly all of adult female mortality is human-caused [66]), both elasticity and variability can be high. Therefore, the negative correlation between the elasticity and variance of a demographic rate may not hold in harvested populations, because artificial mortality patterns differ from natural selection [67,68]. Also, although prime-age female survival might be lower in harvested populations [3], it should be of high importance for population growth.

We found that fecundity rates were lower during the subperiod with high hunting pressure. This could be an unexpected indirect negative effect of hunting on the population. Female brown bears, when with cubs, have been shown to avoid males during the mating season as a counterstrategy to SSI [69,70]. They do so by avoiding good habitats and selecting for habitat in proximity of humans [69], which has a negative effect on their diet quality [71] and could ultimately reduce their subsequent reproductive output [31]. Therefore, as an increase in hunting pressure seems to lead to higher risk of SSI [12,35,36], it could also lead to increased avoidance of males by females with cubs, and lower fecundity. On the other hand, population density generally increased in our study area from 1990 to 2011 [40,41], and density dependence effects may also have resulted in lower fecundity rates in the later period (2006–2011). There is evidence for a decrease in the mean litter size (with more females now having singletons) and an increase in the interlitter interval (with more females weaning their young at 2.5 years old rather than at 1.5 years old) in the latter years of the study (Scandinavian Brown Bear Research Project 1985–2011, unpublished data). Moreover, as we used a pre-breeding census, fecundity rates included the survival of the female to the next census (see Methods). Therefore, a part (between 11% and 18%) of the decrease in fecundity rates observed in this study can be explained by the decrease in survival rates.

Not surprisingly, survival rates of most age classes were lower under high hunting pressure, with the exception of yearlings. Yearling survival might have been higher in the high hunting pressure subperiod because females tended to wean their offspring later in recent years (Scandinavian Brown Bear Research Project 1985–2011, unpublished data). Yearlings staying with their mother until they are 2-year-olds have higher survival than independent yearlings, partly because they are protected from hunting [66]. Cubs are also protected from hunting, but the lower cub survival under high hunting pressure might have reflected increased SSI, caused by an increase in male turnover with the increase in hunting pressure [12,35]. The increase in SSI in the high hunting pressure subperiod might also be influenced by the increase in the proportion of males harvested during this period (57% males in the harvest in 2006–2011 compared with 52% in 1990–2005). In addition, increased density could have negatively affected cub survival by increasing food competition [36]. Density might lower cub survival particularly as it has been found to positively affect the frequency of infanticide [72,73]. Furthermore, although we have no evidence of possible density effects on the survival rates of subadults and adults in our population, and density effects on adult survival are unlikely in large mammals [42], we are unable to exclude the possibility that changes in density may affect the survival rates of all age classes in the population.

Hunting pressure had substantial effects on bear population dynamics; at low hunting pressure, the population appeared to be growing (*λ* = 1.082, 95% CI = 1.052–1.119), but this population trend changed to a decline (*λ* = 0.975, 95% CI = 0.914–1.011) during the period of high hunting pressure. Therefore, if hunting pressure remains the same, the population should, on a long-term scale, decline by about 2% annually. However, the Swedish brown bear population is large, with an estimated 3298 individuals in 2008 [41]. The current management goal in Sweden is to maintain the number of bears on a national level, but allow it to increase or decrease on local scales [41]. As such, the population should be closely monitored to ensure that hunting in the study area does not cause an important decline in the area or a larger-scale decline in the population.

Elasticities were similar in both subperiods as well as in the global study period. This result was expected, as elasticites represent the intrinsic dependence of *λ* to each demographic rate [54]. However, the results of the retrospective analysis differed among periods. At low hunting pressure, the fecundity of adult females explained more of the variation in *λ* than their survival. This pattern was reversed under high hunting pressure, where the survival of adult females explained more of the variation in *λ* than their fecundity. This effect was caused by an increase in the variance of the survival rates, which is expected with an increase in hunting pressure and mortality, but also owing to the decrease in the variance of the fecundity rates. Our results show that harvesting has the potential to severely affect the way a population is regulated. Moreover, this suggests that population growth is mostly driven by recruitment when hunting-induced mortality is low. This prediction is supported by the observation that cub survival explained more variation in population growth under low hunting pressure than under high hunting pressure.

One of our goals was to evaluate the importance of cub survival for population growth to test whether SSI can affect population dynamics. We found that cub survival was relatively important for population growth, with the third highest elasticity, and survival of cubs explained almost as much variation in population growth as the survival of young adult females. When calculated for the entire study period, cub survival explained 16.8% of the variation in *λ*. Considering that 80.9% of the cub mortality occurs during the mating season, and that most, if not all, of this mortality is due to SSI [27], then our results suggest that SSI may explain up to 13.6% of the variation in the population growth rate during our study period (1990–2011). If SSI had not been present (i.e. no cub mortality during the mating season), and everything else being equal, cub survival would have been 80.9% higher (i.e. around 0.968) during 2006–2011. According to our matrix model, increasing cub survival by 80.9% would increase *λ* by 8.17% or 0.080, making *λ* = 1.055 in 2006–2011. This suggests that, even under high hunting pressure, the population would have increased in the absence of SSI. Therefore, male behaviour seems to have an important effect on population dynamics of Scandinavian brown bears.

It has been suggested that human-induced mortality may not be additive to natural mortality, as some compensatory effects might take place [74,75]. As human-induced mortality typically decreases population size, there might be a density-dependent response, increasing natural survival or reproductive rates owing to lower food competition [74,75]. Given that both survival and reproductive rates were lower during the high hunting pressure period, our results indicated that there was no compensatory response to hunting through reproduction in our study population. Bischof *et al.* [43] also found that there was no evidence of compensatory effects of hunting on other sources of mortality in our population. Strong compensation can rarely be expected in long-lived mammals [76]. However, our study supports the contention that hunting can have additional indirect negative effects on populations of large carnivores through SSI [4,21]. As there is evidence that the behaviour of infanticide can be heritable [77,78], this could lead to eco-evolutionary feedbacks on population dynamics. In fact, a reduction in the density of individuals in the population could be a selective pressure to increase SSI as mates become harder to find. Also, an increase in the prevalence of SSI in the population could amplify the decline of the population.

Our study shows that behaviour of individuals and the social biology of a species have important effects on population growth and can interact with hunting mortality to create additional negative effects on the population. Therefore, these factors should be considered when establishing harvest quotas and management policies.

## Supplementary Material

Electronic Supplementary Material

## Data accessibility

The datasets supporting this article have been uploaded as part of the electronic supplementary material.
