# Supplementary material for: The relative importance of direct and indirect effects of hunting mortality on the population dynamics of brown bears
Source: Proc Biol Sci. 2015 Jan 7;282(1798):20141840. doi: 10.1098/rspb.2014.1840 (PMC4262167; doi:10.1098/rspb.2014.1840)
Supplement: Electronic Supplementary Material [file rspb20141840supp1.docx]

## Electronic Supplementary Material

*The relative importance of direct and indirect effects of hunting mortality on the population dynamics of brown bears*

*Gosselin J., Zedrosser A., Swenson, J.E and Pelletier, F.*

**1. Sensitivity analysis**

Of the 183 female bears that were followed during 1990-2011, we lost contact with 26 that were never recaptured or recovered as dead. We calculated the demographic rates of the female component of the population with all of the information available, i.e., including those 26 females for the years they were followed. We had no reason to believe that the survival and reproduction of those 26 females would differ from the population mean after they were lost. However, we performed a sensitivity analysis to ensure that the inclusion or exclusion of these females in our analysis did not impact our results greatly. We calculated the demographic rates under three scenarios: (1) assuming that the 26 females died at the moment they were lost; (2) assuming that the 26 females had a 100% survival probability once lost (until age 24, the maximum observed age of a female in the population); (3) without those 26 females. We carried out these calculations for each study period (1990-2011, 1990-2005 and 2006-2011). The difference between the demographic rates used in our analyses and the demographics rates calculated under the three different scenarios varied between 0 and 7%. We assume that these differences would not cause important differences in the results or conclusions. Therefore, we used demographic rates calculated with all of the information available, i.e., including those 26 females for the years they were followed.

**Table S1** Values of the different demographic rates under four treatments applied for the 26 females with right truncated life histories (lost and never recovered). Normal: using the information available for the years they were followed. S0: assuming they had a null survival once lost. S100: assuming that they had a 100% survival probability once lost. Without: removing these 26 females from the analysis. Each panel represents a different study period.

| **1990-2011** | | | | |
| --- | --- | --- | --- | --- |
| Demographic rate | Normal | S0 | S100 | Without |
| Cub survival | 0.588 | 0.588 | 0.588 | 0.588 |
| Yearling survival | 0.791 | 0.774 | 0.796 | 0.754 |
| 2 y.o. survival | 0.840 | 0.800 | 0.850 | 0.812 |
| 3 y.o. survival | 0.938 | 0.864 | 0.947 | 0.930 |
| 4 to 7 y.o survival | 0.902 | 0.871 | 0.922 | 0.893 |
| 8 y.o. survival | 0.917 | 0.917 | 0.939 | 0.913 |
| 9 to 24 y.o survival | 0.842 | 0.836 | 0.874 | 0.841 |
| 3 y.o. fecundity | 0.166 | 0.152 | 0.167 | 0.164 |
| 4 to 8 y.o. fecundity | 0.488 | 0.474 | 0.500 | 0.484 |
| 9 to 23 y.o. fecundity | 0.502 | 0.499 | 0.521 | 0.502 |
| **1990-2005** | | | | |
| Demographic rate | Normal | S0 | S100 | Without |
| Cub survival | 0.614 | 0.614 | 0.614 | 0.614 |
| Yearling survival | 0.756 | 0.739 | 0.761 | 0.703 |
| 2 y.o. survival | 0.887 | 0.846 | 0.896 | 0.86 |
| 3 y.o. survival | 0.96 | 0.889 | 0.966 | 0.955 |
| 4 to 7 y.o survival | 0.937 | 0.919 | 0.945 | 0.932 |
| 8 y.o. survival | 0.897 | 0.897 | 0.906 | 0.893 |
| 9 to 24 y.o survival | 0.871 | 0.865 | 0.884 | 0.87 |
| 3 y.o. fecundity | 0.194 | 0.18 | 0.195 | 0.193 |
| 4 to 8 y.o. fecundity | 0.559 | 0.55 | 0.564 | 0.556 |
| 9 to 23 y.o. fecundity | 0.589 | 0.585 | 0.598 | 0.588 |
| **2006-2011** | | | | |
| Demographic rate | Normal | S0 | S100 | Without |
| Cub survival | 0.535 | 0.535 | 0.535 | 0.535 |
| Yearling survival | 0.864 | 0.844 | 0.867 | 0.85 |
| 2 y.o. survival | 0.763 | 0.725 | 0.775 | 0.743 |
| 3 y.o. survival | 0.903 | 0.824 | 0.917 | 0.889 |
| 4 to 7 y.o survival | 0.844 | 0.794 | 0.894 | 0.826 |
| 8 y.o. survival | 0.947 | 0.947 | 0.971 | 0.944 |
| 9 to 23 y.o survival | 0.798 | 0.791 | 0.861 | 0.798 |
| 3 y.o. fecundity | 0.122 | 0.123 | 0.123 | 0.12 |
| 4 to 8 y.o. fecundity | 0.38 | 0.362 | 0.401 | 0.374 |
| 9 to 21 y.o. fecundity | 0.392 | 0.386 | 0.42 | 0.39 |

y.o = years old

**2. Hunting pressure periods**

**Table S2** Highest Calinski-Harabasz (CH) index of all possible chronological groups of annual hunting pressure for a fixed number of subperiods in southcentral Sweden from 1990 to 2011. Higher values of the CH index represent higher between-cluster variance relative to within-cluster variance. Therefore, the most probable number of subperiods with different hunting pressure is 2.

| Number of subperiods | Top CH index |
| --- | --- |
| 2 | 24.202 |
| 3 | 22.782 |
| 4 | 18.327 |
| 5 | 20.303 |

**3. Determination of age classes**

Our analyses are based on the female component of the Scandinavian brown bear population from 1990 to 2011. To determine the age classes to be used in the model, we tested different biological hypotheses about which age classes best explained the survival patterns in the population. Our hypotheses were based on the documented minimum age of primiparity, 4 years in Scandinavia [1]. From 1987 to 2006, 39% of females for which primiparity is known were primiparous at age 4, 50% were primiparous at age 5, and 11% at age 6 [1]. Also, female brown bears are considered to be in their prime age between 9 and 20 years old [2]. Inflection points in reproduction are at 4-5 years old and 28-29 years old [2]. However, few studies have tested the difference in survival among ages of adult bears. Kovach *et al*. [3] arbitrarily separated adults into classes spanning 4 years: 5-9 years old, 10-14 years old, 15-19 years old, and 20-29 years old (with a longer span due to small sample size). They found that the survival of 5-9 years old females was lower than the survival of the other age classes, which were all similar. Based on this, we tested the age classes presented in Table S3 and S4.

**Table S3** Hypotheses tested regarding which age classes best described the survival pattern for subadult female brown bears in southcentral Sweden from 1990 to 2011.

|  | Class 1 | Class 2 | Class 3 | Class 4 |
| --- | --- | --- | --- | --- |
| Hypothesis 1 | 0 to 3 |  |  |  |
| Hypothesis 2 | 0 | 1 to 3 |  |  |
| Hypothesis 3 | 0 | 1 | 2-3 |  |
| Hypothesis 4 | 0 | 1 | 2 | 3 |

**Table S4** Hypotheses tested regarding which age classes best described the survival pattern for adult female brown bears in southcentral Sweden from 1990 to 2011.

|  | Class 1 | Class 2 | Class 3 |
| --- | --- | --- | --- |
| Hypothesis 1 | 4 to 20+ |  |  |
| Hypothesis 2 | 4 to 8 | 9 to 20+ |  |
| Hypothesis 3 | 4 to 8 | 9 to 19 | 20+ |
| Hypothesis 4 | 4 to 19 | 20+ |  |
| Hypothesis 5 | 4 to 6 | 7 to 19 | 20+ |
| Hypothesis 6 | 4 to 6 | 7 to 20+ |  |

We included all cubs of the year and all marked females (≥1 year old) for which we had survival information. We had a total of 466 cubs and 180 marked females followed for a total of 901 years. For sample size reasons (see Table S6), all females from 20 to 24 years old were pooled as 20+. Model selection was based on Akaike's information criterion corrected for small sample sizes (AICc). All the models tested were of the form : Survival~Age. All models had a binomial error structure, because survival was 0 or 1. Age was either a continuous variable or a factor of multiple levels, i.e. separated into age classes. We also tested Survival ~1 as a control. Results from the model selection are presented in Table S5.

**Table S5** Performance of the 21 models tested to explain the survival pattern of female brown bears in southcentral Sweden from 1990 to 2011. All models tested were binomial, and were of this form: Survival~Age, where age was either a continuous or separated into age classes. K is the number of estimated parameters for each model; AICc, the information criteria; AICcWt, the Akaike weights (indicate the level of support of any given model being the most parsimonious); Cum.Wt, the cumulative Akaike weights, and LL the log-likelihood of each model.

| # | Model | K | AICc | | Delta_AICc | | AICcWt | | Cum.Wt | | LL |
| --- | --- | --- | --- | --- | --- | --- | --- | --- | --- | --- | --- |
| 1 | 0/1/2/3/4-8/9-19/20+ | 7 | 1348.88 | 0 | | 0.29 | | 0.29 | | -667.4 | |
| 2 | 0/1/2/3/4-8/9-20+ | 6 | 1349.6 | 0.71 | | 0.2 | | 0.49 | | -668.77 | |
| 3 | 0/1/2/3/4-19/20+ | 6 | 1350.42 | 1.54 | | 0.13 | | 0.62 | | -669.18 | |
| 4 | 0/1/2-3/4-8/9-19/20+ | 6 | 1351.32 | 2.44 | | 0.08 | | 0.7 | | -669.63 | |
| 5 | 0/1/2/3/4-6/7-19/20+ | 7 | 1351.64 | 2.76 | | 0.07 | | 0.77 | | -668.78 | |
| 6 | 0/1/2-3/4-8/9-20+ | 5 | 1352.04 | 3.16 | | 0.06 | | 0.83 | | -671 | |
| 7 | 0/1/2/3/4-20+ | 5 | 1352.77 | 3.89 | | 0.04 | | 0.87 | | -671.36 | |
| 8 | 0/1/2-3/4-19/20+ | 5 | 1352.86 | 3.98 | | 0.04 | | 0.91 | | -671.41 | |
| 9 | 0/1/2/3/4-6/7-20+ | 6 | 1353.36 | 4.48 | | 0.03 | | 0.94 | | -670.65 | |
| 10 | 0/1-3/4-8/9-19/20+ | 5 | 1354.31 | 5.43 | | 0.02 | | 0.96 | | -672.13 | |
| 11 | 0/1-3/4-8/9-20+ | 4 | 1355.03 | 6.15 | | 0.01 | | 0.98 | | -673.5 | |
| 12 | 0/1/2-3/4-20+ | 4 | 1355.22 | 6.34 | | 0.01 | | 0.99 | | -673.59 | |
| 13 | 0/1-3/4-19/20+ | 4 | 1355.85 | 6.97 | | 0.01 | | 1 | | -673.91 | |
| 14 | 0/1-3/4-20+ | 3 | 1358.21 | 9.33 | | 0 | | 1 | | -676.1 | |
| 15 | age+age² | 3 | 1366.01 | 17.12 | | 0 | | 1 | | -679.99 | |
| 16 | 0-3/4-8/9-19/20+ | 4 | 1413.66 | 64.78 | | 0 | | 1 | | -702.82 | |
| 17 | 0-3/4-8/9-20+ | 3 | 1414.39 | 65.51 | | 0 | | 1 | | -704.19 | |
| 18 | 0-3/4-19/20+ | 3 | 1415.21 | 66.33 | | 0 | | 1 | | -704.6 | |
| 19 | 0-3/4-20+ | 2 | 1417.57 | 68.69 | | 0 | | 1 | | -706.78 | |
| 20 | age | 2 | 1434.81 | 85.93 | | 0 | | 1 | | -715.4 | |
| 21 | 1 | 1 | 1481.79 | 132.91 | | 0 | | 1 | | -739.89 | |

Three models seem to be equivalent (ΔAICc 0-2). We followed the parsimonious principle and decided to use model #2, because it has fewer parameters than model #1, as adding parameters increases the risk of errors. Moreover, there were only 6 individuals in the 20+ age class (Table S6), which diminished the relevance of estimating demographic rates for this age class.


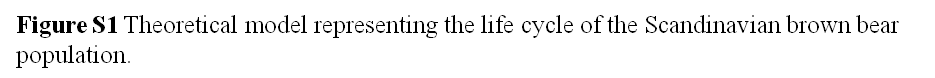

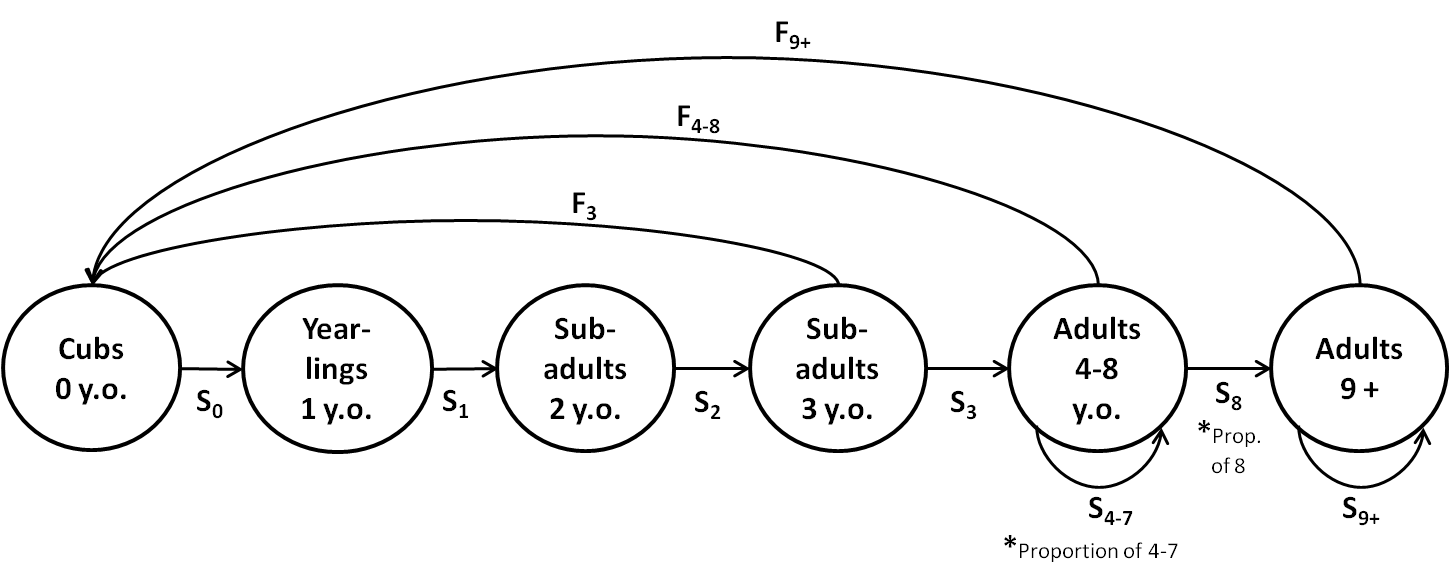
**4.** **Sample size**

**Table S6** Sample sizes for survival information collected from 466 cubs and 180 marked females (≥1 year old) brown bears in southcentral Sweden from 1990 to 2011. Sample sizes are separated into the two different subperiods of hunting pressure.

| Age | Low hunting pressure  (1990-2005) | High hunting pressure  (2006-2011) | Global study period (1990-2011) |
| --- | --- | --- | --- |
| 0 | 311 | 155 | 466 |
| 1 | 90 | 44 | 134 |
| 2 | 62 | 38 | 100 |
| 3 | 50 | 31 | 81 |
| 4 | 50 | 29 | 79 |
| 5 | 40 | 26 | 66 |
| 6 | 35 | 22 | 57 |
| 7 | 33 | 19 | 52 |
| 8 | 29 | 19 | 48 |
| 9 | 26 | 21 | 47 |
| 10 | 23 | 18 | 41 |
| 11 | 20 | 13 | 33 |
| 12 | 18 | 8 | 26 |
| 13 | 15 | 10 | 25 |
| 14 | 12 | 11 | 23 |
| 15 | 11 | 7 | 18 |
| 16 | 11 | 6 | 17 |
| 17 | 8 | 6 | 14 |
| 18 | 7 | 5 | 12 |
| 19 | 5 | 3 | 8 |
| 20 | 4 | 2 | 6 |
| 21 | 4 | 2 | 6 |
| 22 | 4 | 1 | 5 |
| 23 | 1 | 1 | 2 |
| 24 | 1 | 0 | 1 |

**Table S7** Sample sizes for reproductive information collected from 178 marked females (≥1 year old) brown bears in southcentral Sweden from 1990 to 2011. Sample sizes are separated into the two different subperiods of hunting pressure.

| Age | Low hunting pressure  (1990-2005) | High hunting pressure  (2006-2011) | Global study period  (1990-2011) |
| --- | --- | --- | --- |
| 4 | 42 | 26 | 68 |
| 5 | 33 | 22 | 55 |
| 6 | 30 | 18 | 48 |
| 7 | 30 | 14 | 44 |
| 8 | 26 | 14 | 40 |
| 9 | 20 | 18 | 38 |
| 10 | 20 | 16 | 36 |
| 11 | 17 | 12 | 29 |
| 12 | 16 | 8 | 24 |
| 13 | 11 | 10 | 21 |
| 14 | 9 | 10 | 19 |
| 15 | 9 | 6 | 15 |
| 16 | 9 | 5 | 14 |
| 17 | 6 | 6 | 12 |
| 18 | 5 | 5 | 10 |
| 19 | 3 | 3 | 6 |
| 20 | 3 | 1 | 4 |
| 21 | 3 | 1 | 4 |
| 22 | 3 | 1 | 4 |
| 23 | 1 | 0 | 1 |
| 24 | 1 | 0 | 1 |

**5. Confidence interval of population growth (λ) estimates**


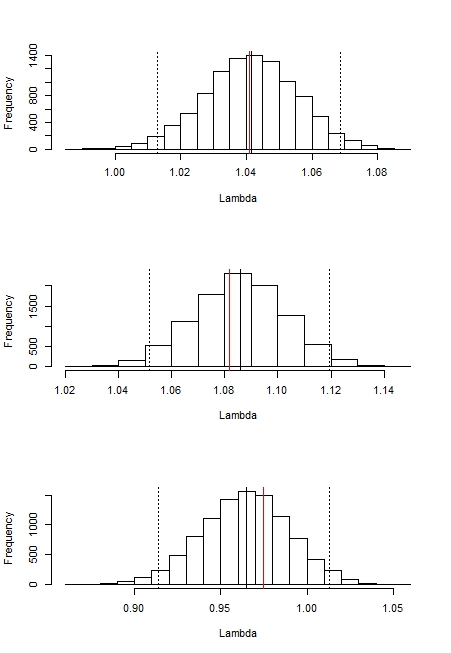


(c)

(b)

(a)

**Figure S2** Bootstrap (10 000 repetitions) of the population growth rate, lambda (λ), in the Scandinavian brown bear population for (a) 1990 to 2011, (b) 1990 to 2005, and (c) 2006 to 2011. Dashed lines represent the 95% confidence limits using the 2.5 and 97.5 percentiles. Black solid lines represent the median of the distribution and the red solid lines represent the real values of λ obtain from our models.

**6. Detailed results of the demographic analyses in the two different hunting pressure subperiods**

**Table S8** Means, standard errors, elasticities, variances and the retrospective analysis results of demographic rates for different age classes of female brown bears in southcentral Sweden. The results of the retrospective analysis show the proportion (%) of the variation in λ that is explained by the variation in each demographic rate. Each panel corresponds to different analyses carried out for different time frame: the low hunting pressure subperiod from 1990-2005 and the high hunting pressure subperiod from 2006-2011.

|  |  |  |  |  |  |
| --- | --- | --- | --- | --- | --- |
| **1990-2005** | | | | | |
| Demographic rate | Mean | Standard error | Elasticity | Variance | Retrospective analysis |
| Cub survival | 0.614 | 0.028 | 0.105 | 0.238 | 18.756 |
| Yearling survival | 0.756 | 0.046 | 0.105 | 0.187 | 9.735 |
| 2 y.o. survival | 0.887 | 0.041 | 0.105 | 0.102 | 3.849 |
| 3 y.o. survival | 0.960 | 0.028 | 0.099 | 0.039 | 1.122 |
| 4 to 8 y.o survival | 0.930 | 0.019 | 0.311 | 0.065 | 19.198 |
| 9 to 24 y.o survival | 0.871 | 0.026 | 0.168 | 0.113 | 11.258 |
| 3 y.o. fecundity | 0.194 | 0.064 | 0.006 | 0.356 | 0.958 |
| 4 to 8 y.o. fecundity | 0.559 | 0.051 | 0.059 | 0.777 | 22.856 |
| 9 to 23 y.o. fecundity | 0.589 | 0.060 | 0.041 | 0.961 | 12.268 |
| **2006-2011** | | | | | |
| Demographic rate | Mean | Standard error | Elasticity | Variance | Retrospective analysis |
| Cub survival | 0.535 | 0.040 | 0.101 | 0.250 | 14.626 |
| Yearling survival | 0.864 | 0.052 | 0.101 | 0.121 | 2.707 |
| 2 y.o. survival | 0.763 | 0.070 | 0.101 | 0.186 | 5.339 |
| 3 y.o. survival | 0.903 | 0.054 | 0.096 | 0.090 | 1.682 |
| 4 to 8 y.o survival | 0.861 | 0.032 | 0.295 | 0.120 | 24.371 |
| 9 to 23 y.o survival | 0.798 | 0.038 | 0.207 | 0.162 | 18.127 |
| 3 y.o. fecundity | 0.122 | 0.054 | 0.005 | 0.168 | 0.432 |
| 4 to 8 y.o. fecundity | 0.380 | 0.054 | 0.050 | 0.579 | 16.571 |
| 9 to 21 y.o. fecundity | 0.392 | 0.058 | 0.046 | 0.716 | 16.145 |

**7. References**

1. Zedrosser A., Dahle B., Støen O.G., Swenson J.E. 2009 The Effects of Primiparity on Reproductive Performance in the Brown Bear. *Oecologia* **160**(4), 847-854. (doi:10.1007/s00442-009-1343-8).

2. Schwartz C.C., Keating K.A., Reynolds H.V., III, Barnes V.G., Jr., Sellers R.A., Jon E.S., Miller S.D., McLellan B.N., Keay J., McCann R., et al. 2003 Reproductive Maturation and Senescence in the Female Brown Bear. *Ursus* **14**(2), 109-119.

3. Kovach S.D., Collins G.H., Hinkes M.T., Denton J.W. 2006 Reproduction and Survival of Brown Bears in Southwest Alaska, USA. *Ursus* **17**(1), 16-29.
